# Small group learning/assessment sessions: A method using test enhanced learning to increase engagement in a basic medical science neuroanatomy course

**DOI:** 10.1002/ca.23839

**Published:** 2022-02-04

**Authors:** Michael F. Nolan, John P. McNamara

**Affiliations:** ^1^ Department of Basic Science Education Virginia Tech Carilion School of Medicine Roanoke Virginia USA

**Keywords:** active learning, medical education, neuroanatomy, self‐assessment

## Abstract

Previous research has shown that test‐enhanced learning with structured feedback facilitates durable learning. We describe a small group learning/assessment activity using these approaches intended to increase engagement and engagement with the course material. We divided our class into six groups of seven students each that worked together in the activity. During each weekly session, course related multiple choice questions were projected and each group instructed to work independently to arrive at a consensus answer for each question. After each question is considered, a faculty facilitator then randomly selects one group to share their choice with the other groups and provide and rationale for their choice. A different group or groups are then called upon to share their choice. When differences emerge, the instructor then facilitates discussion among the groups in an effort to resolve confusion or incomplete or incorrect understanding that becomes evident. We found that attendance for these sessions was greater than for the more traditional lecture based session also included in the course and that students were actively engaged in this learning activity. The success of the small group learning/assessment session is dependent on several factors including the difficulty of the questions and their relatedness to the course objectives, the timing and placement of the session or sessions within the course and the skill of the faculty facilitator in encouraging active participation while ensuring a safe environment in which students can openly share their sometimes incomplete or incorrect understanding of the material.

## 1 INTRODUCTION

Recent studies have shown that testing is an effective method to improve learning (Roediger III & Karpicke, 2006a, 2006b). Several studies have shown that the use of testing results in greater retention and retrieval effectiveness than re‐studying a body of material, a phenomenon known as the “testing effect” (Roediger III & Karpicke, 2006a, 2006b; Glover, 1989; Carrier & Pashler, 1992). Test‐enhanced learning has also been shown to be effective in facilitating long‐term retention of learned material (Karpicke & Roediger III, 2007). Other studies have found that feedback following the test enhances the benefit of testing by correcting errors and reinforcing correct understanding (Butler & Roediger III, 2008; Kang et al., 2007; Pashler et al., 2005).

The greatest benefit of using testing to enhance learning occurs when questions that require effortful retrieval are used (Karpicke & Roediger III, 2008). In addition, research has shown that tests that involve recall (i.e., short answer) promote better and more durable learning than tests that require recognition (i.e., multiple choice tasks) (Butler & Roediger III, 2007; McDaniel, Anderson, et al., 2007; McDaniel, Roediger III, & McDermott, 2007). Furthermore, frequent testing prompts students to keep up with the material and this also contributes to better learning (Fitch et al., 1951). Finally, several investigators have demonstrated that repeated (distributed) testing is more effective than single testing (Dempster, 1998; Karpicke & Roediger III, 2007; Landauer & Bjork, 1978).

Based on reports demonstrating the effectiveness of test‐enhanced learning used together with structured feedback, we sought to incorporate these methods in a learning activity intended primarily to increase student engagement with the course material in a student‐centered, small group learning activity. Our belief was that the use of a teaching approach of demonstrated value would be viewed by the students as engaging and helpful, and would be characterized by increased class attendance and interaction, thereby reversing recent trends of decreasing class participation. To that end we developed a series of small group learning/assessment (SGL/A) activities incorporating retrieval practice with structured feedback that we included in our preclinical neuroanatomy course.

We describe the structure and format of this learning activity and how we incorporated it into our preclinical neuroanatomy course. We describe the materials we created, how they are used and the roles of the faculty in ensuring the success of the activity. We include student comments obtained from the institutionally administered end of course evaluation pertaining to these sessions and report changes in class attendance associated with these sessions. Importantly, we identify and provide recommendations for addressing challenges associated with the use of this type of learning activity in both small class and large class settings.

## 2 MATERIALS AND METHODS

We divided our class of 42 students into six groups of seven students each with each group being charged to work together to answer STEP 1 type questions. Each group was provided with five (5) large (8½ × 11) laminated cards of the same color representing their group on which were printed in large font one of the letters A through E. One individual in each group was designated as “group leader”; this individual being responsible for holding up a particular card when called upon.

Eight, 1‐h SGL/A sessions were scheduled, one at the end of each week during the 8‐week course. Sessions were scheduled at the end of a series of topically related learning activities, typically on the last scheduled class day of the week. Topics addressed during each session were those that were considered since the previous SGL/A session.

During each SGL/A session, vignette structured multiple choice questions (MCQ's) were projected onto a screen visible to each group. Students were instructed to discuss and debate the question within their group and come to consensus regarding a best response to the question. After a reasonable amount of time, the instructor called upon the “group leader” of each group to simultaneously raise a card representing the answer choice of that group. Cards were to be displayed such that only the instructor could see the choice. The class as a whole was instructed to NOT look at answers chosen by other groups and it is important that students adhere to this request. The instructor then recorded the response choice of each group and instructed that the cards be lowered and placed face down after the response of each group was noted.

The instructor then called upon a particular group to indicate their answer to the question under study and provide a rationale for their choice. The instructor then called upon another group to indicate their answer and to explain why they chose their answer. Since the faculty instructor knew the answers of each group, the second group called upon could be chosen from those that chose a different answer from the first group or the same answer. Strategies used to determine which group to call upon first and subsequently will be discussed below.

When groups indicated different answers, discussion was prompted between the involved groups, and other groups as well, in an attempt to identify areas of incomplete or incorrect understanding or to clarify concepts or correct errors in understanding that became evident. The responsibility of the faculty facilitator was to monitor the inter‐group interaction to ensure accuracy and avoid the introduction of confusing or incorrect information. Important also, was the need to maintain a collegial and professional interaction among students and an environment where students felt safe in possibly exposing their lack of understanding or familiarity with the material. This can be a challenging role for the faculty instructor and must be effectively managed for the session to be perceived as valuable. The intended end point is to ensure that all students developed a clear understanding of the specific content embedded in the question. This process had to be carefully managed so that the incorrect thinking by students offering explanations did not confuse other students who were not participating in the discussion. The decision as to when to terminate discussion and to project the question with the correct answer and then to move on to the next question is one of the most challenging tasks for the faculty instructor.

Following faculty‐monitored discussion, most groups came to agreement on a particular answer for each question. At this point, the instructor then projected the question with the correct answer indicated and asked if there were any questions about the subject of the question that still remained. If so, those questions were addressed briefly. The instructor then advanced to the next question. SGL/A class sessions were not mandatory nor were other components of the course such as lectures. Neither individual nor group performance on the questions included in the sessions was used in determining a students' grade in the course.

Attendance at the SGL/A sessions and at other scheduled class sessions (lectures) was collected and used as a measure of student engagement. Student feedback regarding these sessions was obtained by means of a single narrative question included in the institutionally standardized end of course survey.

This study (IRB #19‐1097) was determined to be exempt by the Institutional Review Board at Virginia Polytechnic Institute & State University (Virginia Tech).

## 3 RESULTS

Attendance at 6 of 8 SGL/A sessions was 42/42 students (100%) with attendance numbers of 40/42 (95%) and 41/42 (98%) for the remaining two sessions, with all absences having been administratively approved. In contrast, attendance at the more traditional class sessions (lectures) ranged from 16 to 32 (38%–76%) of students attending class depending on the faculty presenter and the proximity of the class to the end of course examination.

An average of 10–20 questions were considered and discussed during each session. Most students did not comment on this issue; however, a few, as will be noted below, would have preferred that more questions would have been discussed.

Most students responding to the survey indicated that they appreciated the SGL/A sessions and found them valuable as a method of review and preparation for the course summative examination. Many students offered suggestions for improvement. Below is a sample of student comments and suggestions. They were selected to represent both the strengths and weaknesses of the sessions.

Good! The discussion in choosing an answer and then explaining the right/wrong answers is helpful. Seems a little technical sometimes. Sometimes goes a little slowly.

I liked them a lot; make sure the questions at SGLA are not primarily fact based though since those don't need much discussion.

I really liked the SGLA sessions! They definitely exposed me to the types of questions that would be asked on the exams and were a great way to synthesize the material learned over the week.

That was great for my learning. It is always beneficial to have practice questions to get a better idea of what you know as well as preparing me for the exam. It also encourages me to stay on top of the material so that I can feel I can contribute to my group. I would recommend this for every course but I know that there can be difficulties with scheduling.

Questions were very helpful, but I wish we went through more questions per session.

The weakness was that after attending a few sessions, I realized that the intergroup discussion component went for way too long such that we could only get through 8 or 10 questions at the most.

SGLA sessions were a great "self‐checks." I found these sessions helpful to constantly remind myself of important information throughout the block. Great way to review material in a practical way.

The SGL/A sessions were not helpful. We spent too much time "discussing" questions. If we all got the same right answer. I feel that it is not necessary to go over ALL of the answer choices and discuss them all.

It would more effective if the SGL/A session quizzed the PREVIOUS week's materials instead of the CURRENT week's material. This would allow ample time for the student to prepare and study instead of merely relying on short‐term memory and recall.

The SGLA sessions were great (I really liked them and wouldn't get rid of them), but every person felt we needed more questions. A solution to this would be to keep the SGLA sessions as is and then provide a minimum of 10 questions (with answer explanations) to do on our own. This would increase the number of questions, which was a major criticism of the sessions, and it would give everyone an opportunity to do some questions alone, which is the way we are tested (we don't get to work in groups for our final block exams).

## 4 DISCUSSION

Student attendance in non‐mandatory class activities is generally dependent on a number of factors. Some students attend class as a matter of habit, others as a reflection of their learning styles (auditory learners) and still others because they recognize and value the experience and expertise of the faculty. Attendance and participation is generally a reflection of their perception of the value of that activity. Our finding that student attendance and engagement in these activities over other class activities suggests that the SGL/A sessions were considered valuable by the students.

We expected and observed that most of the students in each group actively participated in the intra‐group discussions aimed at arriving at a consensus answer for each question. We noted that depending on the subject of the question, different students within a group took a leadership role in the discussion, suggesting that some students may have felt more confident in discussing a particular topic than others. Differences of opinion as to the intended group answer were raised and discussed in a respectful manner even when it appeared that a consensus could not be achieved. In these instances, decision by majority vote among the group was used. We were careful to not call on individual students, but rather on the group so to avoid calling attention to students who may not be as confident as some of their peers, either because they did not understand the material or simply had not studied that material by the time of the session. We realized most importantly that by calling upon specific students to respond to a question, we would have likely caused some students to not attend these sessions for fear of being put in an uncomfortable position.

Discussion within the group was robust as evidenced by the fact that for many questions, time for group discussions had to be limited in order to reveal the correct answer and respond to groups that might have chosen an incorrect response. As described below, permitting groups to present and defend their answer choice before revealing the correct answer was key in determining whether the concepts addressed in each question were understood or not. In addition, allowing students themselves to correct errors in understanding among their classmates resulted in the type of cognitive engagement we were trying to promote.

A review of the free comments provided by the students obtained at the end of the course dealt primarily with suggestions for increasing the value of the sessions. A common theme to emerge dealt with the number of questions considered during a particular session. The most frequent suggestion was to increase the number of questions considered, an objective that could be accomplished within the allocated class time by limiting the time used for discussing issues such why an incorrect response was incorrect. We have found that taking time to provide a brief explanation of why incorrect choices are incorrect allows students to revise their understanding in the event it resulted in the selection of an incorrect answer. We discuss this important aspect of session management below.

The use of formative assessment techniques is not new in preclinical medical education. Our goal was to develop a class activity would engage the students and promote active participation. We wanted to develop an activity that students would feel comfortable with, engage in and provide meaningful feedback regarding their accomplishments and would be viewed as valuable in assisting them achieve their learning objectives. We report that the vast majority of the eight scheduled SGL/A sessions were attended by all 42 students and that most students within a group participated in the discussions of their group. We also found that all groups were engaged and participated on multiple occasions during the intergroup discussions.

The question of the adaptability of this learning activity to larger classes is a challenging one. Our experience is with a class size of 42 students that we divided into six groups of seven students each. With this arrangement we were able to monitor intra‐group discussions and ensure that over a 50‐min class period, we were able to maintain student engagement.

For schools with larger numbers of students we would recommend dividing the class into sections of no more than approximately 50 students that can be divided into smaller groups and create several sections, each section with its own faculty facilitator. In groups of more than 7–8 students the less confident or unprepared students might feel marginalized and hesitant to contribute. With more than six groups, some groups may not have the opportunity to enter into the intergroup interactions and feel that they were not given the chance to fully engage in the learning activity.

Only a relatively small number of the questions we specifically wrote for this project were used during each session. Most students did not comment on this issue; however, a few did indicate that they would have preferred that more questions would have been discussed. It is likely that these students were among those with a better understanding of the material and needed less time to arrive at the correct answer to a particular question. We determined that the number of questions that could be addressed in each session depended on several factors, one of which was the complexity of the stem and the plausibility of the answer choices. The more data in the vignette and the more detailed and complicated the information, the more time was necessary for each group to arrive at a consensus answer. An additional factor was how much time the faculty facilitator allowed for discussion when groups differed in their opinion regarding the correct answer. Careful monitoring and facilitating of the intergroup interactions was necessary to ensure that all students acquired a correct and satisfactory understanding of the subject of the question. This important faculty responsibility had to take into account the current fund of knowledge of the students at the time of the session and the logic and understanding of the material represented in the explanations provided for selecting a particular answer. When incorrect or incomplete thinking contributed to the selection of an incorrect answer, time was needed to assist the student or group in correcting or redirecting their thinking in the proper direction.

While we developed this activity for use in a neuroanatomy course, we recognize that that this approach is easily adaptable to other preclinical courses such as gross anatomy, histology or embryology. We are currently developing questions to be used in similar sessions in our preclinical gross anatomy course.

We found to use of colored cards to be more convenient and equally effective as the use of a “clicker” system. Student responses were easily observed by a quick visual scan of the room and no time was required to reset the equipment when moving on to the next question. Colored cards involve essentially no additional cost to the course and are easily transported to settings where this activity might be scheduled.

The success and perceived value of the SGL/A activities as described here rests heavily on several factors including the type of follow‐up questions asked by the instructor, the order in which specific group are called upon to defend their choice, the accuracy of the rationales provided by each group, the difficulty of particular questions, the importance the students attach to the activity in terms of grade weight and the extent to which the instructor might have to guide or shape the interactive discussions among the groups. Our experience suggests that the SGL/A sessions described here effectively promote student engagement in important learning and self‐assessment activities.

Because we divided our students into small groups or “teams”, we were careful to use the designation SGL/A so as to avoid any confusion with a different approach known as Team Based Learning (TBL). The sessions described here are not to be confused with other forms of team based learning. Among other major differences, TBL as commonly understood and described in the literature utilizes individual readiness and group readiness tests while ours does not. Our sessions, were initially developed to enhance class participation and engagement with the course material.

## 5 RECOMMENDATIONS FOR SUCCESSFUL IMPLEMENTATION

Based on comments from students and discussion among the participating faculty, we offer the following recommendations to ensure a successful and effective use of this approach.

### 5.1 Ensure that the questions are sufficiently challenging (but not overly difficult) so that weaknesses or deficiencies in understanding can be highlighted and corrected

We wrote topic related, vignette based multiple‐choice questions of the STEP 1 format. Most of the questions required students to utilize history and physical examination data to answer higher order questions such as “which of the following would most likely be found on further examination?” and “which of the following antiepileptic drugs would be most effective for use with this patient?” In the case of the first question, the students must first localize the lesion producing the signs and symptoms presented in the stem and then recognize clinical findings associated with disease or injury affecting other nearby neural structures. In the case of the later questions, all five of the drugs listed are antiepileptic drugs and the students is expected to consider multiple factors in making a decision as to which drug would be best in this situation.

Questions that are too easy do not challenge the students. When all groups select the correct answer, the incentive for students to reflect on the scope and depth of their own knowledge is reduced. The only option left to the instructor is to ask groups to explain why one or more of the incorrect distractors is incorrect or not the best choice. While certainly helpful, most students would rather move on to another question that might better help identify an actual deficiency in their learning. Many students seem to prefer confirmation of what they know rather than identification of what they do not know. Make sure all students are satisfied before moving on to the next question.

Questions that are too difficult may convey a set of faculty expectations that are beyond the scope of the course. The use of too many questions that students are unable to answer correctly may lead to frustration and anxiety that can impair further learning. When multiple sessions are scheduled within a course, questions that are seen to be too difficult for a particular session during the course may persuade some students, possible incorrectly, that they are lagging behind, not spending enough time with the material or are inefficient with their study habits. Frustration may lead some students to stop attending these sessions.

Related to the issue of question difficulty is the matter of faculty content expertise. Higher order, thought provoking questions will, during both the intra‐group discussions and the inter‐group interactions, bring to light areas of incomplete understanding or misunderstanding of subject matter. Faculty leading these sessions must be able to quickly recognize the indicators of these problems and respond by identifying, clarifying and resolving these problems before they derail forward progress. For example, a student who is uncertain about a particular topic may hear and understand as correct, the comments or explanations of another student who may also have an incorrect view of the issue. Faculty must be able to recognize when this occurs and intervene quickly, before some students become more confused than before the discussion began. There is a real risk of failure if participating faculty are unable to deal confidently and correctly with the frequently unanticipated gaps or errors in student knowledge that are brought to light during the class activity.

Learning is strengthened through effortful retrieval and this requires that one's understanding is reasonably challenged and that questions must be constructed to provide these types of cognitive challenges.

### 5.2 Clearly define the topics that will be addressed during each session

It is important that faculty clearly define the topics that will be considered during each session. This helps students to focus their study and not spend valuable time on topics that will not be necessary in order to understand the questions in a particular session. Depending on the number of sessions embed in the course; each session need not be limited to content considered since the previous session. For courses comprised of serially ordered units, each depending on the previous unit, it might be desirable to state that each session may or will include topics from each previous unit of the course. In this way, knowledge can be developed and expanded in a more realistic way that is more applicable to the overall subject of the course. Only the final session of the course may be viewed as comprehensive of the entire course.

### 5.3 Vary the approach to determining which group to call upon to indicate their answer choice and offer a rationale

When selecting a particular group to indicate their answer and discuss their rationale it is essential to vary whether the group called upon first chose the correct or an incorrect answer. If an instructor consistently calls first upon a group that selected an incorrect answer, the class will soon recognize this pattern and any group called upon first will immediately experience a sudden sense of anxiety. Similarly, if the first group called upon consistently chose the correct answer, then other groups that selected a different answer will likewise experience a sense of anxiety. In either case, emotional reactions based on a perception of what answer might be the correct one will invariably affect, generally negatively, any discussion likely to follow. Thus, it is important for the facilitator to vary in a non‐predictable way which groups are called upon to provide an answer and explanation to a particular question.

### 5.4 Schedule multiple session within the course rather that one at the end of the course

Because these are teaching sessions as well as opportunities for student self‐assessment, they should not be viewed as time lost from formal instruction. Students might initially view these class sessions narrowly for the purpose of providing feedback and as a measure of progress though out the course. However, it is important to emphasize and demonstrate through facilitated discussion and active group participation that an important goal is fundamental learning and reinforcement of previously learned material. By scheduling multiple session, typically at the end of a series of related instructional units, both students and faculty can monitor progress as well as ensure that material considered early in a course that might be foundational, is well understood before more complicated topics are studied at a later time. Because these sessions are instructional as well as assessment in nature and occur at multiple time during the course, students need not feel that they need to “cram” or spend excessive amounts of time preparing for each session. By design, learning will occur as a result of participation, whether active or passive.

### 5.5 Require each respondent to be clear and complete as possible when responding to a question or when explaining an answer

Students differ in their ability to accurately and concisely express their views. Incomplete or unclear responses may further confuse any already confused or ill‐informed students. Be patient when listening to a student explanation and when necessary, ask for clarification when you think other students or groups might be helped by some clarifying comments or explanatory information. In this regard, when a student may be struggling to express their question or point of view clearly, it is often helpful to respectfully rephrase the question and ask if this better captures the student's intent.

### 5.6 Respect the wishes of students who may not wish to be called upon

Some students are by nature quiet, reserved or even shy. Students who may be struggling with material during the course or who may lack the confidence that familiarity or facility with the material would bring, may understandably be uncomfortable with being brought into the spotlight among their peers. Many of these students may be more participatory in their own small group, but feel less comfortable in front of a larger group. It is important to the success of the session that the preferences of these students be recognized and respected. Do not force a student to expose their uncertainty or lack of confidence with the material. It is essential that students be made to feel safe in exposing their ignorance or uncertainty. Failure on the part of the faculty to maintain a safe environment can severely undermine not only the effectiveness of these sessions but also the success of a particular student.

### 5.7 Resist the temptation to “speed through” the question pool

The main objectives of these interactive sessions are to allow students to uncover deficiencies or errors in their knowledge and to facilitate a student‐driven process aimed at remediation, knowledge development and reinforcement. The impetus to move quickly through a pool of questions without discussion frequently arises when the questions, or a series of questions are judged to be too easy; that is, when all groups arrive at the same answer in a short span of time. Students see easy questions as representing material they are already familiar with and time spent reviewing this material may be seen as time not well spent. When viewed this way, many students would prefer more “doses” of positive reinforcement than fewer during a particular session. Students are less likely to push for more questions when they are challenged or unsure and discover that, through discussion and the sharing of ideas, their knowledge becomes more solidified and retrievable.

### 5.8 Encourage all members of the group to participate in the group discussions

Students must be convinced that gaps in their knowledge or incomplete understanding must be identified before they can be remedied. Open and full discussion among a small group of peers allows for uncovering these weaknesses. Members of the group should be encouraged to questions the belief of their group peers in a professional and constructive manner. Students can be reminded that the ability to explain a concept, process or principle is good evidence of a firm knowledge and understanding of that material. Group members who do not actively participate is group discussions deprive themselves of this important form of self‐assessment.

We counsel against deferring to the smartest or most confident student in the group. Even the best‐informed students sometimes have gaps in their knowledge.

### 5.9 Consider writing your own question

This recommendation may be difficult to achieve. The advantage of developing your own question pool is that questions can be appropriately linked to your specific course content, and of a depth and scope you expect of the students. Students invariably expect that whatever is embedded in a course, particularly what can be viewed as “practice questions”, should contribute to success on the summative course examination. Questions that are not perceived to be tightly linked to course content may inadvertently distract some students leading to inefficient, inappropriate or ineffective study efforts. In addition, when content and knowledge is built progressively during the course, question difficulty and complexity can be fitted to the understanding students will be expected to demonstrate at specific intervals. This is an important consideration when multiple sessions are scheduled within a course. The challenge is in writing questions that are not so easy that all groups select the correct answer, but rather challenge students to question their understanding regarding a particular topic.

### 5.10 Consider framing these sessions in a game format where successful group work over multiple sessions might result in a group or groups receiving some special recognition for their collaborative work

Students are more engaged when learning is fun. By framing these learning and self‐assessment sessions as components of a knowledge game involving several teams, student's viewpoint is frequently shifted from a task (self‐assessment and learning) that might otherwise be boring to an activity that is more often associated with comradery and enjoyment. One might consider offering a nominal prize such as gift cards for the group with the highest overall score at the end of the course.

A limitation of this work is the limited amount of feedback we were able to obtain from the students. Assessment and evaluation at our school is centrally administrated and we were limited to only one question on our end‐of‐course evaluation survey. An additional limitation is the lack of evidence relating these sessions to durable learning. We recognize the limitations posed by the lack of quantitative data regarding student performance in the course and are considering experimental protocols that will allow us to address this important question.

Our initial efforts however, were directed toward increasing student engagement in a learning activity, believing that engagement (participation) of a sufficient number of students was necessary before we might be able to assess objective learning. The work described here has shown us that almost all of our 42 students participated in the eight sessions and we now feel that we can move forward with designing a formal study to measure actual learning.

## 6 CONCLUSION

The use of test‐enhanced teaching methods has been shown result in durable learning in a variety of educational settings. We developed a series of learning activities involving these methods for use in a small group setting in a preclinical medical neuroanatomy course in an effort to increase engagement with the material as evidenced by increased attendance at these sessions as compared to other class sessions (i.e., lectures) delivered by the faculty. We found that attendance at these sessions was much higher than for lectures, suggesting that the students recognized the value of these sessions. Engagement, both physical and cognitive was observed in the form of robust discussions among students as they worked to arrive at a consensus answer to questions addressing topics in the course. The success of the sessions was dependent on faculty monitoring of the process. As we conducted these sessions during the course, we identified factors that can both contribute to and hinder successful implementation and thus undermine their effectiveness.
